# Supplementary material for: Histone lactylation couples cellular metabolism with developmental gene regulatory networks
Source: Nat Commun. 2024 Jan 2;15:90. doi: 10.1038/s41467-023-44121-1 (PMC10762033; doi:10.1038/s41467-023-44121-1)
Supplement: Supplementary file 1 — Supplementary Information [file 41467_2023_44121_MOESM1_ESM.pdf]

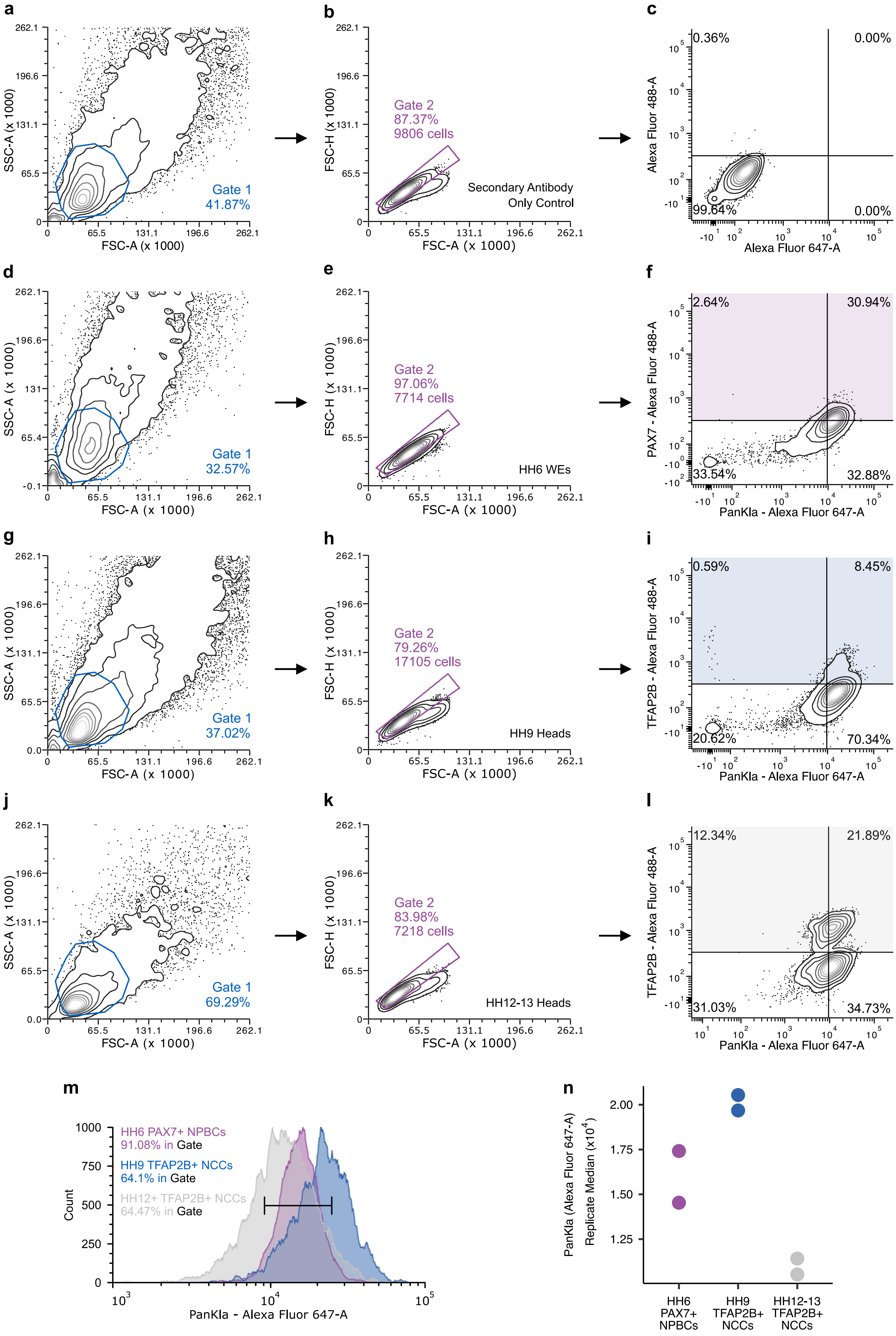
a
SSC-A (x 1000)
FSC-A (x 1000)
Gate 1
41.87%
b
FSC-H (x 1000)
FSC-A (x 1000)
Gate 2
87.37%
9806 cells
Secondary Antibody
Only Control
c
Alexa Fluor 488-A
Alexa Fluor 647-A
0.36%
0.00%
99.64%
0.00%
d
SSC-A (x 1000)
FSC-A (x 1000)
Gate 1
32.57%
e
FSC-H (x 1000)
FSC-A (x 1000)
Gate 2
97.06%
7714 cells
HH6 WEs
f
PAX7 - Alexa Fluor 488-A
PanKla - Alexa Fluor 647-A
2.64%
30.94%
33.54%
32.88%
g
SSC-A (x 1000)
FSC-A (x 1000)
Gate 1
37.02%
h
FSC-H (x 1000)
FSC-A (x 1000)
Gate 2
79.26%
17105 cells
HH9 Heads
i
TFAP2B - Alexa Fluor 488-A
PanKla - Alexa Fluor 647-A
0.59%
8.45%
20.62%
70.34%
j
SSC-A (x 1000)
FSC-A (x 1000)
Gate 1
69.29%
k
FSC-H (x 1000)
FSC-A (x 1000)
Gate 2
83.98%
7218 cells
HH12-13 Heads
l
TFAP2B - Alexa Fluor 488-A
PanKla - Alexa Fluor 647-A
12.34%
21.89%
31.03%
34.73%
m
Count
PanKla - Alexa Fluor 647-A
HH6 PAX7+ NPBCs
91.08% in Gate
HH9 TFAP2B+ NCCs
64.1% in Gate
HH12+ TFAP2B+ NCCs
64.47% in Gate
n
PanKla (Alexa Fluor 647-A)
Replicate Median (x10^4)
HH6 PAX7+ NPBCs
HH9 TFAP2B+ NCCs
HH12-13 TFAP2B+ NCCs

Supplementary Figure 1 – Flow cytometry gating strategy and control plots for lactylation level quantification experiment.

(**A-C**) Contour plots showing the relative density of events in subsequent gating steps for cell samples stained only with secondary antibodies (Alexa647 and Alexa488). Gates are displayed as colored lines and the percentage/number of events in the gate is shown. Fluorescent intensity gates (C) are used for all of the following samples to define specific populations. (**D-F**) Contour plots showing the relative density of events in subsequent gating steps for HH6 samples stained with PAX7 (Alexa488) and PanKla (Alexa647) antibodies. Gates are displayed as colored lines and the percentage/number of events in the gate is displayed. (**G-I**) Contour plots showing the relative density of events in subsequent gating steps for HH9 samples stained with TFAP2B (Alexa488) and PanKla (Alexa647) antibodies. Gates are displayed as colored lines and the percentage/number of events in the gate is shown. (**J-L**) Contour plots showing the relative density of events in subsequent gating steps for HH12-13 samples stained with TFAP2B (Alexa488) and PanKla (Alexa647) antibodies. Gates are displayed as colored lines and the percentage/number of events in the gate is shown. (**M**) Histograms of lactylation levels (Alexa647-A) for cells in the purple (F), blue (I), and gray (L) gates defining PAX7+ NPBCs, TFAP2B+ pre-migratory/delaminating NCCs, and TFAP2B+ migratory NCCs respectively. The percentage of cells in the gate defined by the black lines is displayed. (**N**) Dot plot showing the median of the distribution of lactylation levels for the cells in the purple (F), blue (I), and gray (L) gates for both biological replicates.

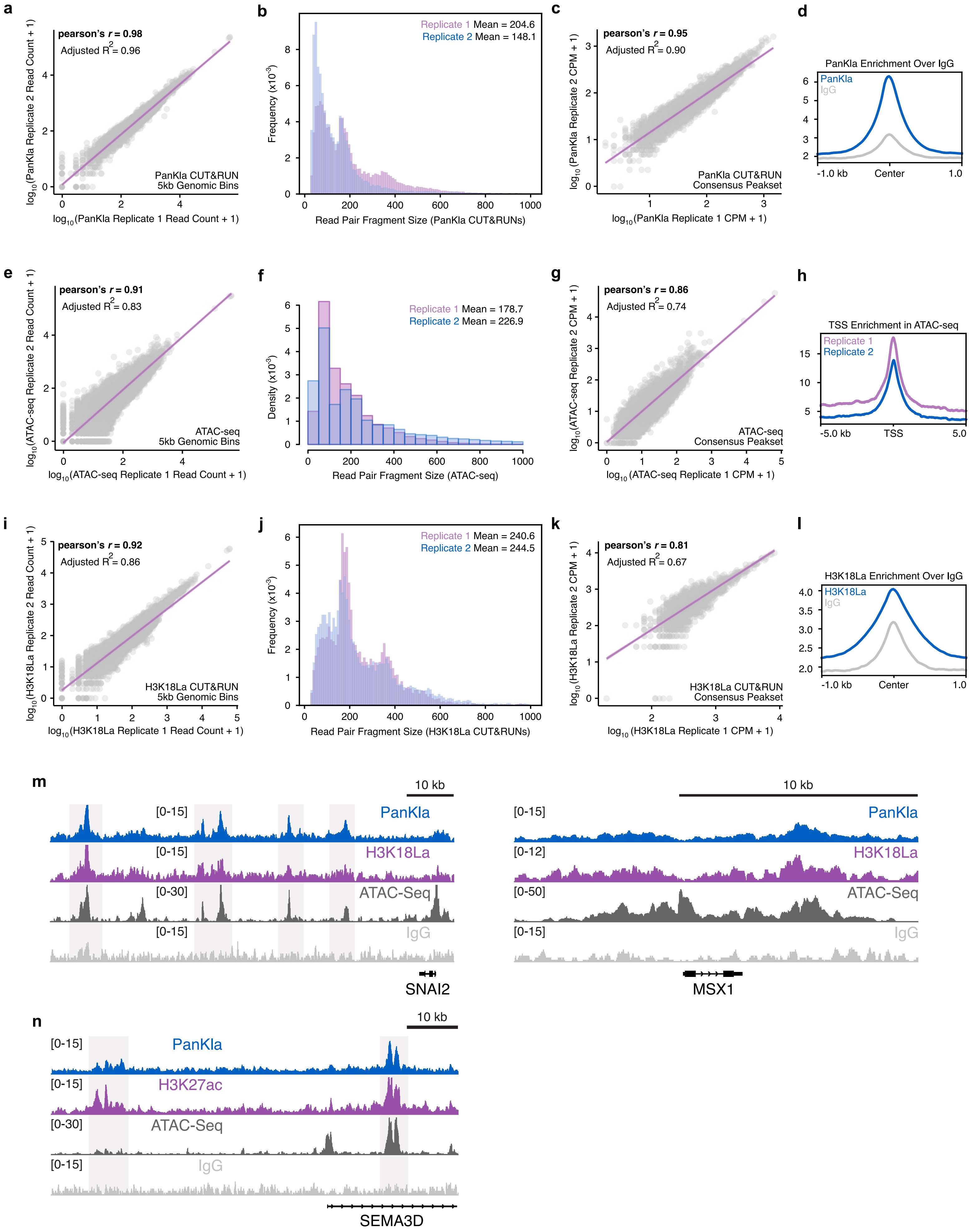
a
pearson's r = 0.98
Adjusted R2 = 0.96
PanKla CUT&RUN
5kb Genomic Bins
log10(PanKla Replicate 1 Read Count + 1)
log10(PanKla Replicate 2 Read Count + 1)
b
Replicate 1 Mean = 204.6
Replicate 2 Mean = 148.1
Frequency (x10-3)
Read Pair Fragment Size (PanKla CUT&RUNs)
c
pearson's r = 0.95
Adjusted R2 = 0.90
PanKla CUT&RUN
Consensus Peakset
log10(PanKla Replicate 1 CPM + 1)
log10(PanKla Replicate 2 CPM + 1)
d
PanKla Enrichment Over IgG
PanKla
IgG
-1.0 kb
Center
1.0
e
pearson's r = 0.91
Adjusted R2 = 0.83
ATAC-seq
5kb Genomic Bins
log10(ATAC-seq Replicate 1 Read Count + 1)
log10(ATAC-seq Replicate 2 Read Count + 1)
f
Replicate 1 Mean = 178.7
Replicate 2 Mean = 226.9
Density (x10-3)
Read Pair Fragment Size (ATAC-seq)
g
pearson's r = 0.86
Adjusted R2 = 0.74
ATAC-seq
Consensus Peakset
log10 (ATAC-seq Replicate 1 CPM + 1)
log10(ATAC-seq Replicate 2 CPM + 1)
h
TSS Enrichment in ATAC-seq
Replicate 1
Replicate 2
-5.0 kb
TSS
5.0
i
pearson's r = 0.92
Adjusted R2 = 0.86
H3K18La CUT&RUN
5kb Genomic Bins
log10(H3K18La Replicate 1 Read Count + 1)
log10(H3K18La Replicate 2 Read Count + 1)
j
Replicate 1 Mean = 240.6
Replicate 2 Mean = 244.5
Frequency (x10-3)
Read Pair Fragment Size (H3K18La CUT&RUNs)
k
pearson's r = 0.81
Adjusted R2 = 0.67
H3K18La CUT&RUN
Consensus Peakset
log10 (H3K18La Replicate 1 CPM + 1)
log10(H3K18La Replicate 2 CPM + 1)
l
H3K18La Enrichment Over IgG
H3K18La
IgG
-1.0 kb
Center
1.0
m
10 kb
[0-15] PanKla
[0-15] H3K18La
[0-30] ATAC-Seq
[0-15] IgG
SNAI2
10 kb
[0-15] PanKla
[0-12] H3K18La
[0-50] ATAC-Seq
[0-15] IgG
MSX1
n
10 kb
[0-15] PanKla
[0-15] H3K27ac
[0-30] ATAC-Seq
[0-15] IgG
SEMA3D

Supplementary Figure 2 – Quality control metrics for PanKla and H3K18La CUT&RUN as well as neural fold ATAC-seq datasets.

(**A**) Scatterplot of read count from each PanKla replicate CUT&RUN alignment file at regions obtained after binning the genome into 5kb fragments (213380 bins). Magenta line is the line of best fit from regression analysis. (**B**) Overlayed histograms of read pair fragment size distribution for both PanKla CUT&RUN replicates. (**C**) Scatter plot of sequencing depth normalized PanKla signal from each replicate at consensus PanKla peakset (10612 peaks). (**D**) Profile plot showing cumulative PanKla and IgG signal at PanKla consensus peakset. PanKla signal displayed as average between replicates. (**E**) Scatterplot of read count from each neural fold ATAC-seq replicate alignment file at regions obtained after binning the genome into 5kb fragments (213312 bins). Magenta line is the line of best fit from regression analysis. (**F**) Overlayed histograms of read pair fragment size distribution for both ATAC-seq replicates. (**G**) Scatter plot of sequencing depth normalized PanKla signal from each replicate at consensus PanKla peakset (38250 peaks). (**H**) Profile plots showing enrichment of ATAC-seq signal at transcription start sites (TSS) for both replicates of each sample. (**I**) Scatterplot of read count from each H3K18La replicate CUT&RUN alignment file at regions obtained after binning the genome into 5kb fragments (213311 bins). Magenta line is the line of best fit from regression analysis. (**J**) Overlayed histograms of read pair fragment size distribution for both H3K18La CUT&RUN replicates. (**K**) Scatter plot of sequencing depth normalized H3K18La signal from each replicate at consensus H3K18La peakset (1578 peaks). (**L**) Profile plot showing cumulative H3K18La and IgG signal at H3K18La consensus peakset. H3K18La signal displayed as average between replicates. (**M**) Genome browser tracks showing PanKla CUT&RUN, H3K18La CUT&RUN, and ATAC-seq peaks at the SNAI2 and MSX1 loci. IgG track included as a control. (**N**) Genome browser tracks showing PanKla CUT&RUN, H3K27ac CUT&RUN, and ATAC-seq peaks at the SEMA3D locus. IgG track included as a control. RefSeq gene annotation tracks are used to visualize genes in genome browser panels. Non-curated non-coding RNA annotations are not displayed.

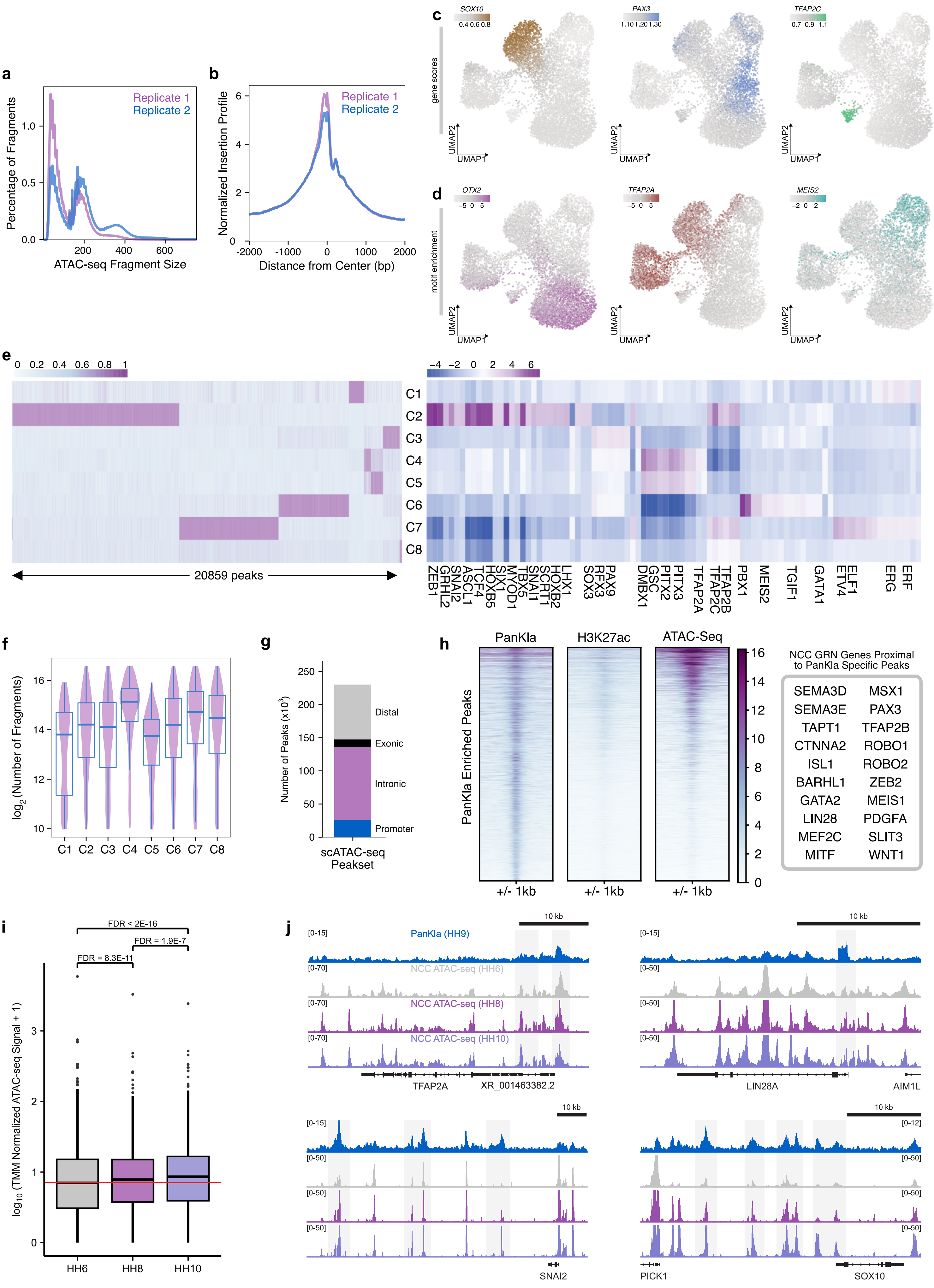
a
Replicate 1
Replicate 2
Percentage of Fragments
ATAC-seq Fragment Size
b
Normalized Insertion Profile
Distance from Center (bp)
c
gene scores
SOX10
PAX3
TFAP2C
UMAP1
UMAP2
d
motif enrichment
OTX2
TFAP2A
MEIS2
e
C1
C2
C3
C4
C5
C6
C7
C8
20859 peaks
ZEB1 GRHL2 SNAI2 ASCL1 TCF4 HOXB5 SIX1 MYOD1 TBX5 SNAI1 SCRT1 HOXB2 LHX1 SOX3 RFX3 PAX9 DMBX1 GSC PITX2 PITX3 TFAP2A TFAP2C TFAP2B PBX1 MEIS2 TGIF1 GATA1 ETV4 ELF1 ERG ERF
f
log2(Number of Fragments)
g
Number of Peaks (x10^3)
Distal
Exonic
Intronic
Promoter
scATAC-seq Peakset
h
PanKla
H3K27ac
ATAC-Seq
PanKla Enriched Peaks
+/- 1kb
NCC GRN Genes Proximal to PanKla Specific Peaks
SEMA3D MSX1
SEMA3E PAX3
TAPT1 TFAP2B
CTNNA2 ROBO1
ISL1 ROBO2
BARHL1 ZEB2
GATA2 MEIS1
LIN28 PDGFA
MEF2C SLIT3
MITF WNT1
i
FDR < 2E-16
FDR = 1.9E-7
FDR = 8.3E-11
log10 (TMM Normalized ATAC-seq Signal + 1)
HH6
HH8
HH10
j
PanKla (HH9)
NCC ATAC-seq (HH6)
NCC ATAC-seq (HH8)
NCC ATAC-seq (HH10)
10 kb
TFAP2A
XR_001463382.2
LIN28A
AIM1L
SNAI2
PICK1
SOX10

Supplementary Figure 3 – Quality control metrics and peak calling parameters for scATAC-seq dataset.

(**A**) Fragment size distribution of biological replicates from the scATAC-seq dataset showing nucleosomal binding pattern. (**B**) Profile plots showing enrichment of signal at transcription start sites (TSS) for both replicates of the scATAC-seq dataset. (**C**) ScATAC-seq UMAPs showing the projection of genescores for the transcription factors Sox10, Pax3, and Tfap2c. (**D**) ScATAC-seq UMAPs showing the motif enrichment projection for transcription factors Otx2, Tfap2a, and Meis2. (**E**) Heatmaps showing the significantly enriched peaks (FDR<0.1, log2FC>2) and associated top enriched motifs across the scATAC-seq clusters. (**F**) Violin plots showing the distribution of unique nuclear fragments per cells after filtering at each cluster in the scATAC-seq dataset. (**G**) Stacked barplot showing the genomic distribution of called peaks in the scATAC-seq dataset. (**H**) Tornado plots showing PanKla, H3K27ac, and bulk ATAC-seq signal at lactylation-enriched peaks used for projection onto the scATAC-seq UMAP in Figure 3C. Box to the right shows the NCC GRN genes that are associated with said lactylation-enriched peaks. (**I**) Boxplot of TMM normalized ATAC-seq signal at lactylated peaks (from the aggregate ATAC peakset) at three developmental stages. Kruskal-Wallis test (χ^2 = 128.51, df = 2, p-value = 1.244058E-28), followed by *ad hoc* pair-wise Wilcoxon rank sum test (n_{HH6} = 10018 peaks, n_{HH8} = 10018 peaks, n_{HH10} = 10018 peaks). The p-value was corrected for multiple comparisons using FDR (HH6 vs HH8 p-value = 8.328737E-11, HH8 vs HH10 p-value = 1.885227E-07, HH6 vs HH10 p-value = 4.637372E-28). Boxplot center line is median, box limits are upper and lower quartiles, whiskers are the 1.5X interquartile range, and individual points are outliers. (**J**) Genome browser tracks showing replicate average ATAC-seq signal from HH6, HH8, and HH10 NCC samples as well as HH9 PanKla at the TFAP2A, LIN28A, SNAI2, and SOX10 genomic loci. RefSeq gene annotation tracks are used to visualize genes in genome browser panels. Non-curated non-coding RNA annotations are not displayed for LIN28A, SNAI2, and SOX10 genomic loci.

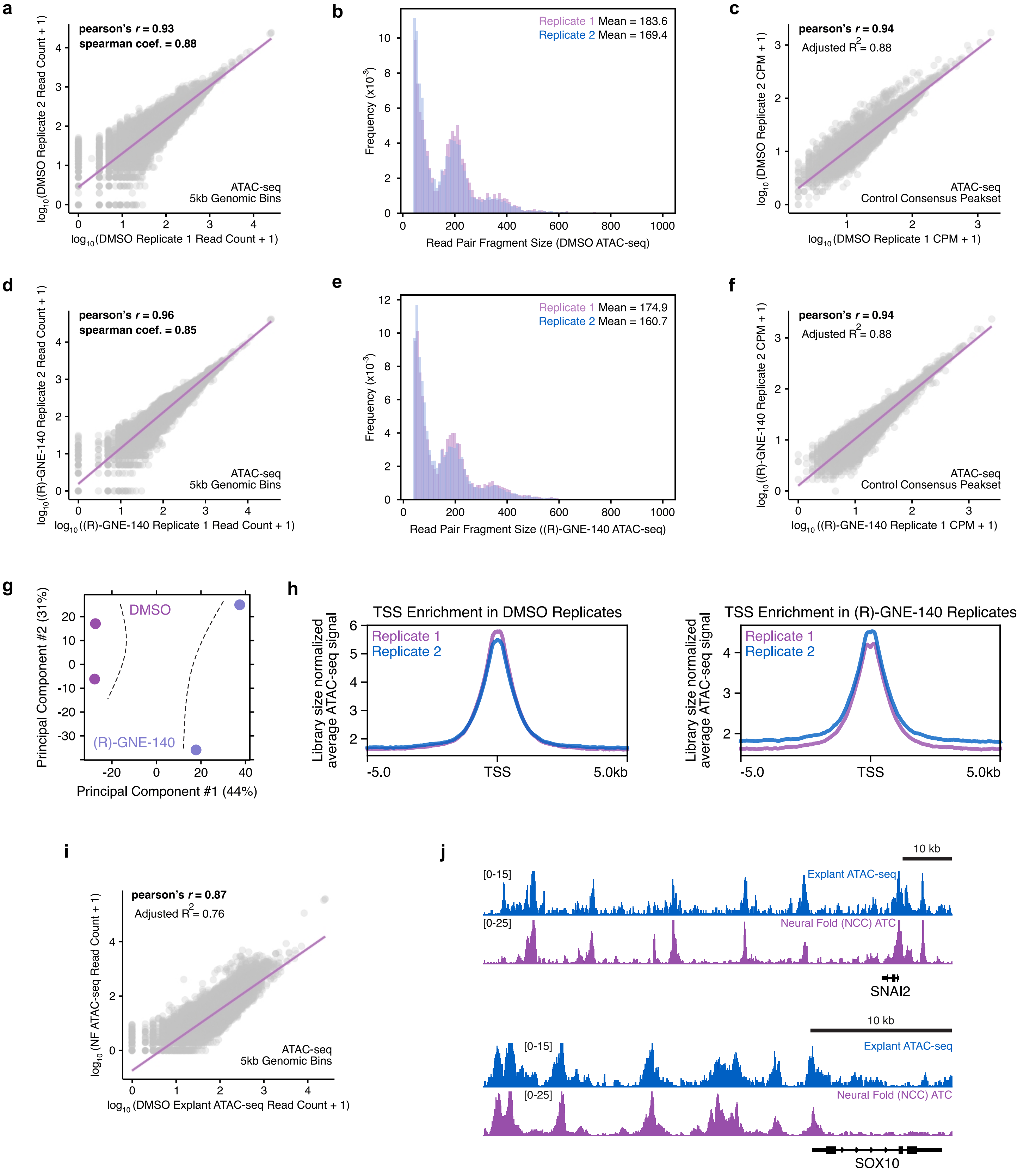
a
pearson's r = 0.93
spearman coef. = 0.88
ATAC-seq
5kb Genomic Bins
log10(DMSO Replicate 2 Read Count + 1)
log10(DMSO Replicate 1 Read Count + 1)
b
Replicate 1 Mean = 183.6
Replicate 2 Mean = 169.4
Frequency (x10^-3)
Read Pair Fragment Size (DMSO ATAC-seq)
c
pearson's r = 0.94
Adjusted R2 = 0.88
ATAC-seq
Control Consensus Peakset
log10(DMSO Replicate 2 CPM + 1)
log10(DMSO Replicate 1 CPM + 1)
d
pearson's r = 0.96
spearman coef. = 0.85
ATAC-seq
5kb Genomic Bins
log10((R)-GNE-140 Replicate 2 Read Count + 1)
log10((R)-GNE-140 Replicate 1 Read Count + 1)
e
Replicate 1 Mean = 174.9
Replicate 2 Mean = 160.7
Frequency (x10^-3)
Read Pair Fragment Size ((R)-GNE-140 ATAC-seq)
f
pearson's r = 0.94
Adjusted R2 = 0.88
ATAC-seq
Control Consensus Peakset
log10((R)-GNE-140 Replicate 2 CPM + 1)
log10((R)-GNE-140 Replicate 1 CPM + 1)
g
DMSO
(R)-GNE-140
Principal Component #2 (31%)
Principal Component #1 (44%)
h
TSS Enrichment in DMSO Replicates
TSS Enrichment in (R)-GNE-140 Replicates
Replicate 1
Replicate 2
Library size normalized average ATAC-seq signal
-5.0
TSS
5.0kb
i
pearson's r = 0.87
Adjusted R2 = 0.76
ATAC-seq
5kb Genomic Bins
log10 (NF ATAC-seq Read Count + 1)
log10 (DMSO Explant ATAC-seq Read Count + 1)
j
10 kb
[0-15]
Explant ATAC-seq
[0-25]
Neural Fold (NCC) ATC
SNAI2
SOX10

Supplementary Figure 4 – Quality control metrics for ATAC-seq performed on DMSO and (R)-GNE-140 treated NCC explant cultures.

(**A**) Scatterplot of read count from each DMSO ATAC-seq replicate alignment file at regions obtained after binning genome into 5 kb fragments (213335 bins). Magenta line is the line of best fit from regression analysis. (**B**) Overlayed histograms of read pair fragment size distribution for both DMSO ATAC-seq replicates. (**C**) Scatter plot of sequencing depth normalized ATAC-seq signal from each replicate at consensus DMSO peakset (40408 peaks). (**D**) Scatterplot of read count from each (R)-GNE-140 ATAC-seq replicate alignment file at regions obtained after binning genome into 5 kb fragments (213335 bins). (**E**) Overlayed histograms of read pair fragment size distribution for both (R)-GNE-140 ATAC-seq replicates. (**F**) Scatter plot of sequencing depth normalized ATAC-seq signal from each replicate at consensus DMSO peakset (4048 peaks). (**G**) Principal Component Analysis (PCA) plot showing replicates of each sample (DMSO and (R)-GNE-140). Principal component #1, accounting for most of the variance, separates the samples by treatment (dashed gray lines). (**H**) Profile plots showing enrichment of ATAC-seq signal at TSS for both replicates of each sample. (**I**) Scatterplot of read count (averaged between replicates) for DMSO and NF ATAC-seq samples at 5 kb genomic bins. (**J**) Genome browser tracks showing ATAC-seq peaks for explant (DMSO) and NF samples at the SNAI2 and SOX10 loci. RefSeq gene annotation tracks are used to visualize genes in genome browser panels. Non-curated non-coding RNA annotations are not displayed.

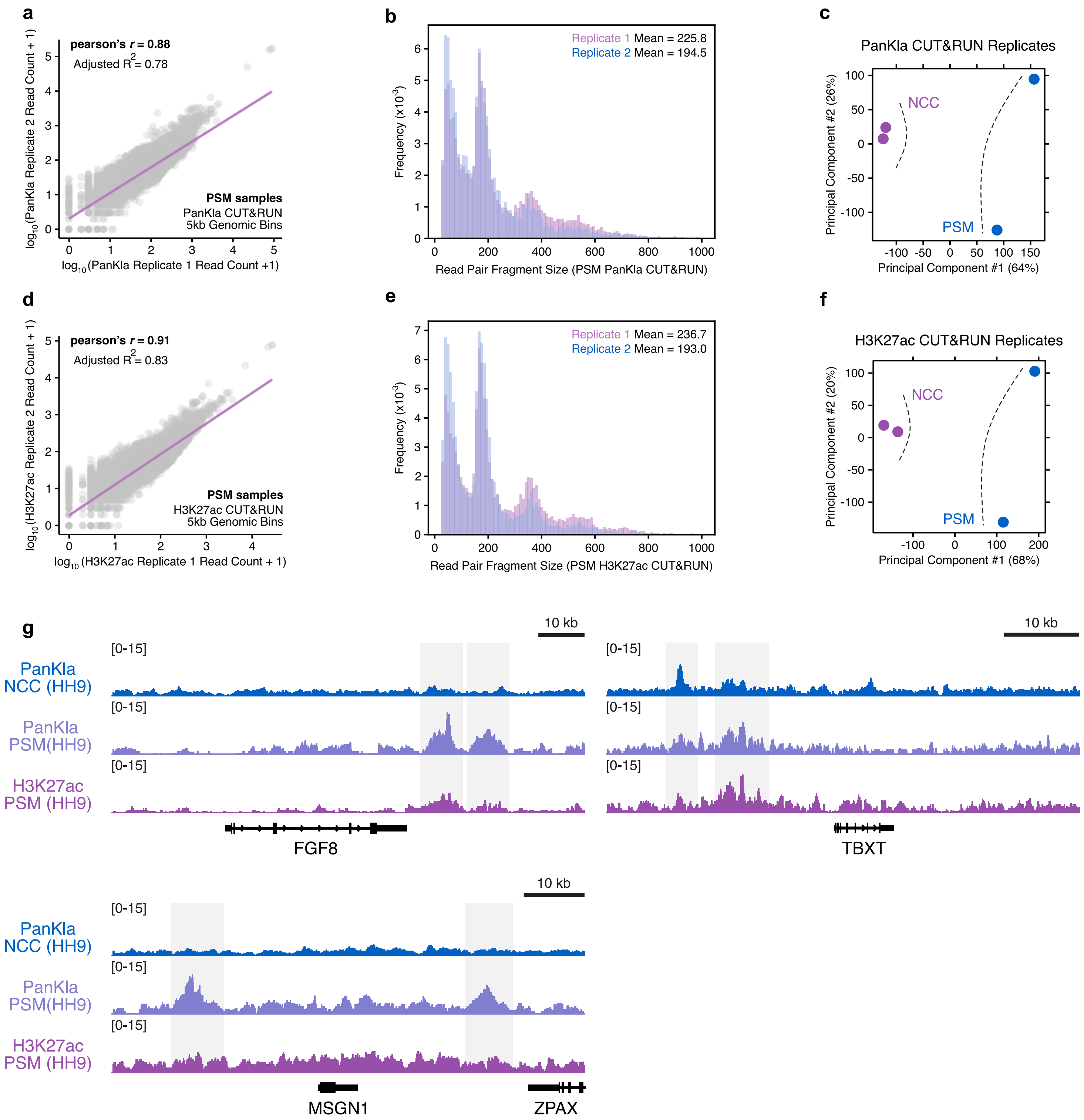
a
pearson's r = 0.88
Adjusted R2 = 0.78
PSM samples
PanKla CUT&RUN
5kb Genomic Bins
log10(PanKla Replicate 1 Read Count +1)
log10(PanKla Replicate 2 Read Count + 1)
b
Replicate 1 Mean = 225.8
Replicate 2 Mean = 194.5
Frequency (x10-3)
Read Pair Fragment Size (PSM PanKla CUT&RUN)
c
PanKla CUT&RUN Replicates
NCC
PSM
Principal Component #1 (64%)
Principal Component #2 (26%)
d
pearson's r = 0.91
Adjusted R2 = 0.83
PSM samples
H3K27ac CUT&RUN
5kb Genomic Bins
log10(H3K27ac Replicate 1 Read Count + 1)
log10(H3K27ac Replicate 2 Read Count + 1)
e
Replicate 1 Mean = 236.7
Replicate 2 Mean = 193.0
Frequency (x10-3)
Read Pair Fragment Size (PSM H3K27ac CUT&RUN)
f
H3K27ac CUT&RUN Replicates
NCC
PSM
Principal Component #1 (68%)
Principal Component #2 (20%)
g
10 kb
[0-15]
PanKla NCC (HH9)
PanKla PSM(HH9)
H3K27ac PSM (HH9)
FGF8
TBXT
MSGN1
ZPAX

Supplementary Figure 5 – Quality control metrics for PanKla and H3K27ac CUT&RUNs performed on PSM cells.

(**A**) Scatterplot of read count from each PanKla replicate CUT&RUN alignment file at regions obtained after binning the genome into 5kb fragments (213312 bins). Magenta line is the line of best fit from regression analysis. (**B**) Overlayed histograms of read pair fragment size distribution for both PSM PanKla CUT&RUN replicates. (**C**) PCA plot comparing PanKla CUT&RUNs performed in NCCs (**Supplementary Figure 1A-1D**) and PSM cells. Principal component #1, accounting for most of the variance, separates the samples by cell type (dashed gray lines). (**D**) Scatterplot of read count from each H3K27ac replicate CUT&RUN alignment file at regions obtained after binning the genome into 5kb fragments (213312 bins). Magenta line is the line of best fit from regression analysis. (**E**) Overlayed histograms of read pair fragment size distribution for both PSM H3K27ac CUT&RUN replicates. (**F**) PCA plot comparing H3K27ac CUT&RUNs from NCCs and ones performed on PSM cells. Principal component #1, accounting for most of the variance, separates the samples by cell type (dashed gray lines). (**G**) Genome browser tracks showing PanKla CUT&RUN for NC and PSM cells as well as H3K27ac CUT&RUN for PSM cells at PSM-specific loci (FGF8, TBXT, and MSGN1).

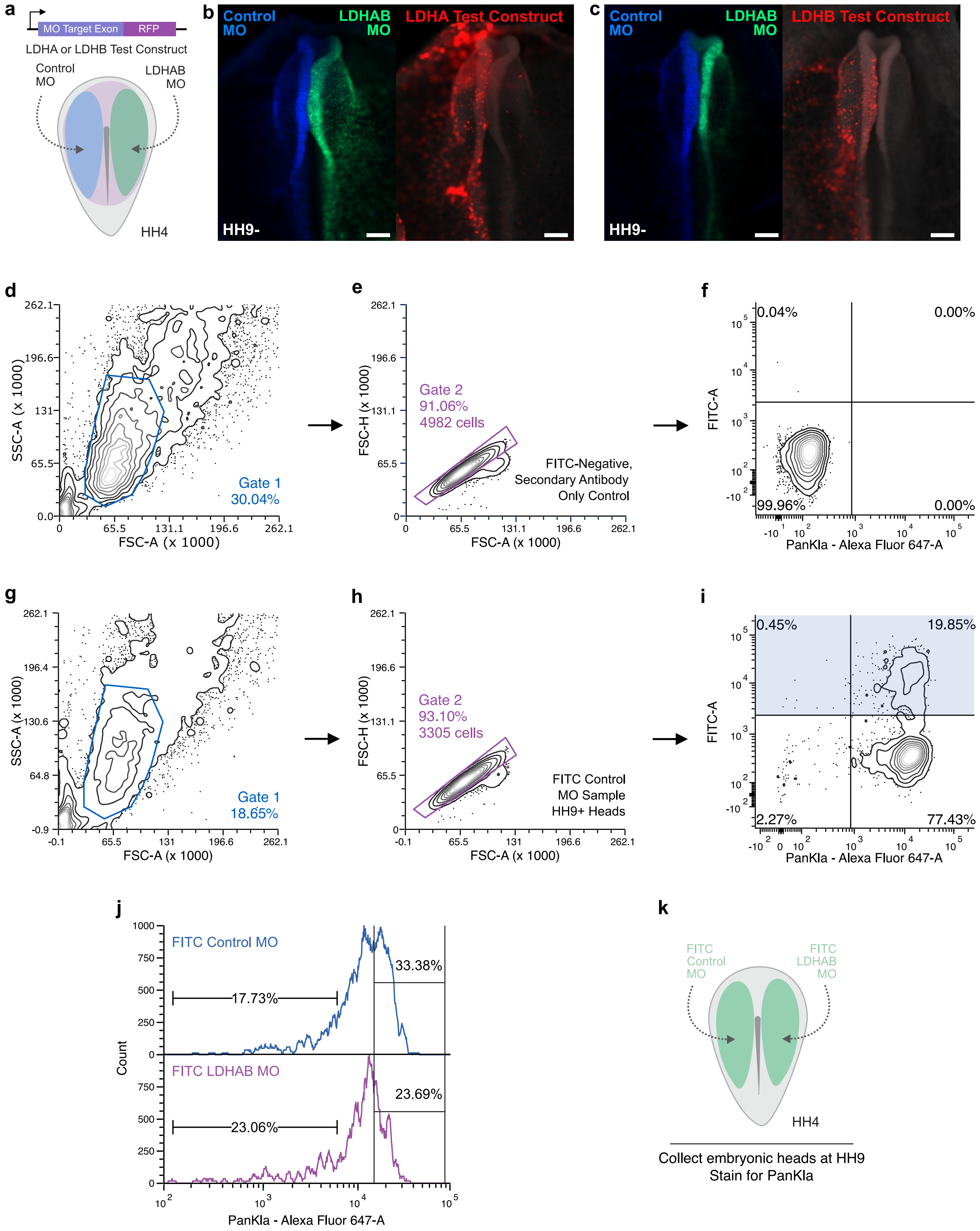
a
MO Target Exon
RFP
LDHA or LDHB Test Construct
Control MO
LDHAB MO
HH4
b
Control MO
LDHAB MO
LDHA Test Construct
HH9-
c
Control MO
LDHAB MO
LDHB Test Construct
HH9-
d
SSC-A (x 1000)
FSC-A (x 1000)
Gate 1
30.04%
e
FSC-H (x 1000)
FSC-A (x 1000)
Gate 2
91.06%
4982 cells
FITC-Negative, Secondary Antibody Only Control
f
FITC-A
0.04%
0.00%
99.96%
0.00%
PanKla - Alexa Fluor 647-A
g
SSC-A (x 1000)
FSC-A (x 1000)
Gate 1
18.65%
h
FSC-H (x 1000)
FSC-A (x 1000)
Gate 2
93.10%
3305 cells
FITC Control MO Sample HH9+ Heads
i
FITC-A
0.45%
19.85%
2.27%
77.43%
PanKla - Alexa Fluor 647-A
j
Count
FITC Control MO
17.73%
33.38%
FITC LDHAB MO
23.06%
23.69%
PanKla - Alexa Fluor 647-A
k
FITC Control MO
FITC LDHAB MO
HH4
Collect embryonic heads at HH9
Stain for PanKla

Supplementary Figure 6 – LDHA/B knockdown validation and flow cytometry gating strategy for lactylation level quantification in LDHA/B morphant and control HH9 embryonic head cells.

(**A**) Diagram depicting experimental strategy for testing the knock-down efficiency of LDHA and LDHB MOs. Embryos are injected one side with LDHA or LDHB test construct mixed with LDHA/B MO mix and on the other side with the same test construct mixed with the Control MO. (**B**) Image of bilaterally transfected HH9 embryo showing loss of RFP expression from the LDHA test construct on the LDHA/B MO transfected side (4/4 embryos display the similar loss of RFP expression). Scale bars represent 200 µm. (**C**) Image of bilaterally transfected HH9 embryo showing loss of RFP expression from the LDHB test construct on the LDHA/B MO transfected side (4/4 embryos display the similar loss of RFP expression). Scale bars represent 200 µm. (**D-F**) Contour plots showing the relative density of events in subsequent gating steps for cell samples stained only with secondary antibody (Alexa647). Gates are displayed as colored lines and the percentage/number of events in the gate is shown. Fluorescent intensity gates (F) are used to define the boundaries of the FITC population. (**G-I**) Contour plots showing the relative density of events in subsequent gating steps for FITC control MO cell samples stained for PanKla (Alexa647-A). Gates are displayed as colored lines and the percentage/number of events in the gate is displayed. The PanKla levels of cells in the blue gate (I) were quantified in FITC control and LDHAB MO samples. (**J**) Histogram of lactylation levels (Alexa647-A) for cells in the blue gate (F) for FITC control MO (blue, top) and LDHAB MO samples (purple, bottom). (**K**) Diagram depicting experimental strategy for quantifying the effects of LDHA/B MO treatment on lactylation levels. Each embryo was transfected with FITC control and LDHA/B MOs at HH4 and allowed to develop until HH9. Embryonic heads were bisected, collected, dissociated, and subjected to cell-suspension staining for PanKla. The cells were then analyzed on an BD FACSCelesta.

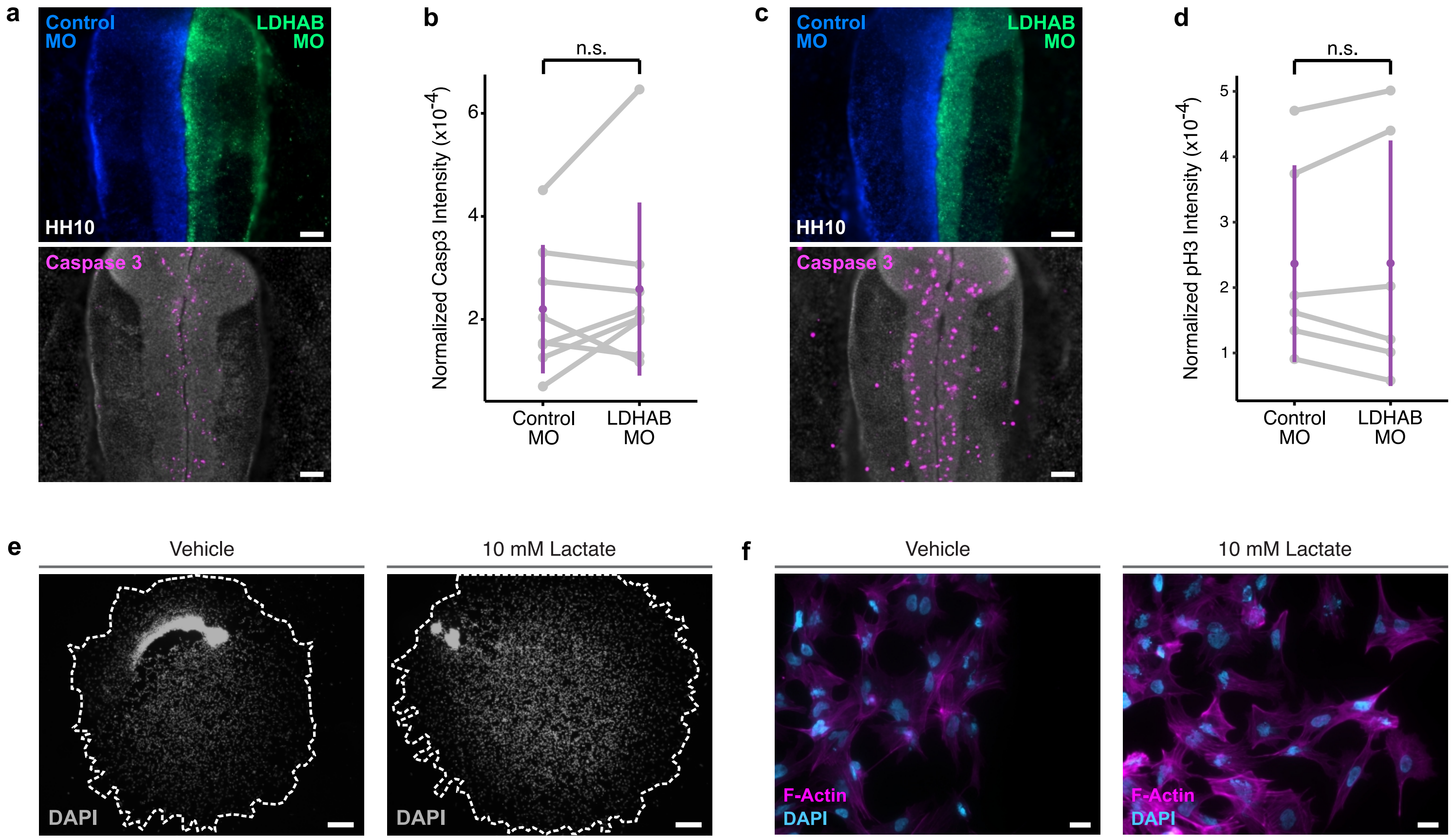

a
Control MO
LDHAB MO
HH10
Caspase 3
b
n.s.
Normalized Casp3 Intensity (x10-4)
2
4
6
Control MO
LDHAB MO
c
Control MO
LDHAB MO
HH10
Caspase 3
d
n.s.
Normalized pH3 Intensity (x10-4)
1
2
3
4
5
Control MO
LDHAB MO
e
Vehicle
10 mM Lactate
DAPI
DAPI
f
Vehicle
10 mM Lactate
F-Actin
DAPI
F-Actin
DAPI

Supplementary Figure 7 – Control experiments for LDHA/B MO and lactate treatments.

(**A**) IF staining for Caspase 3 on embryo transfected with Control and LDHA/B MOs. Scale bars represent 100 µm. (**B**) Paired stripchart showing the quantification of Casp3 fluorescence intensity (normalized by area of measurement) in the Control and LDHA/B MO transfected sides of embryos. Purple bars represent standard deviation. For the analysis, n.s. p-value > 0.05 (p-value = 0.4609375**)**, Wilcoxon signed rank test (n = 8 biological replicates). (**C**) IF staining for 1hosphor Histone H3 on embryo transfected with Control and LDHA/B MOs. Scale bars represent 100 µm. (**D**) Paired stripchart showing the quantification of pH3 fluorescence intensity (normalized by area of measurement) in the Control and LDHA/B MO transfected sides of embryos. Purple bars represent standard deviation. For the analysis, n.s. p-value > 0.05 (p-value = 0.84375), Wilcoxon signed rank test (n = 6 biological replicates). (**E**) Representative images showing whole explants in the vehicle control and 10 mM sodium lactate conditions used for analysis in Figure 4K. Scale bars represent 200 µm. (**F**) Higher magnification images of vehicle and sodium lactate treated cells stained for F-Actin and DAPI to show cellular morphology. Scale bars represent 20 µm.

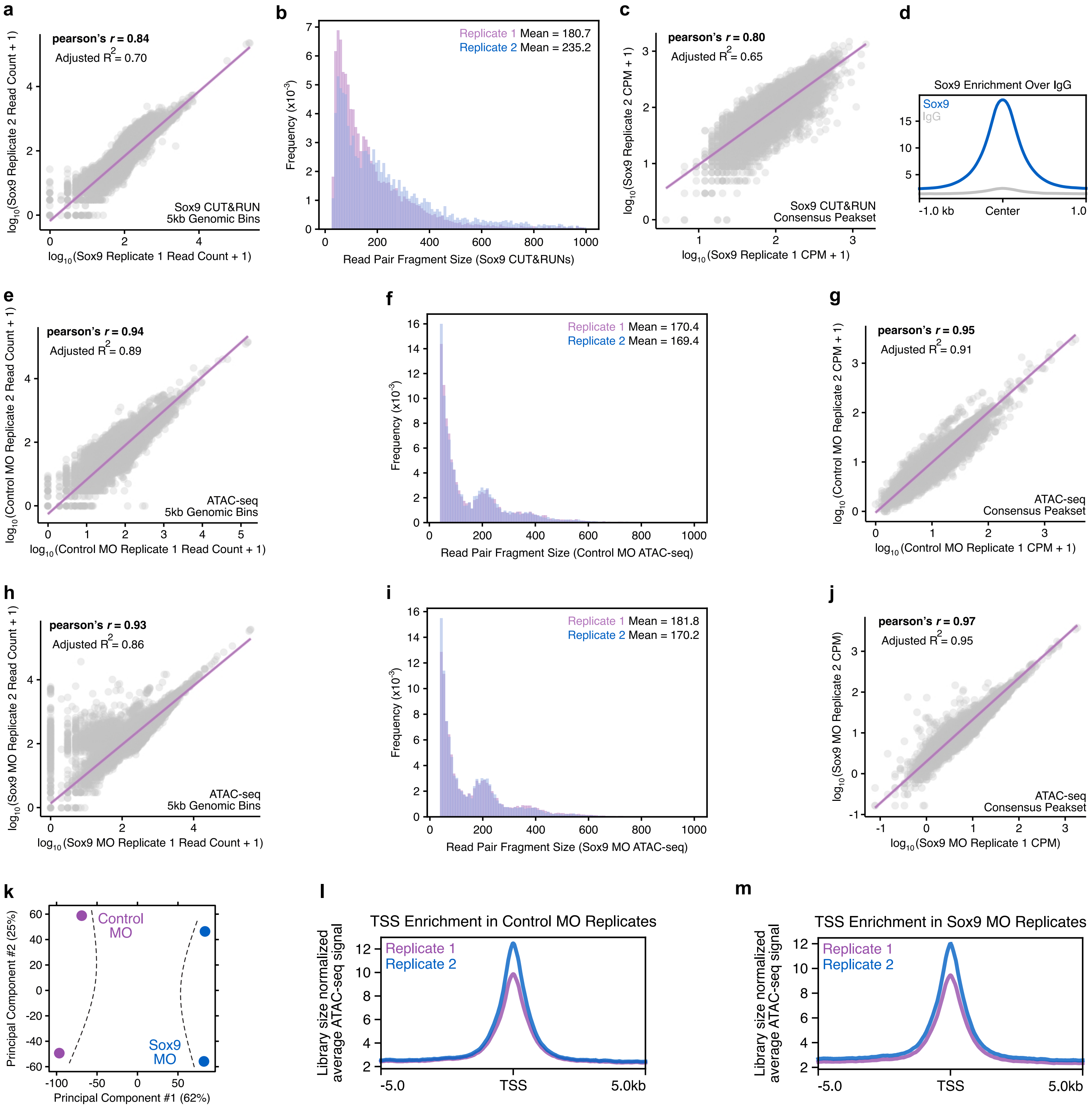

a
pearson's r = 0.84
Adjusted R2 = 0.70
Sox9 CUT&RUN
5kb Genomic Bins
log10(Sox9 Replicate 2 Read Count + 1)
log10(Sox9 Replicate 1 Read Count + 1)
b
Replicate 1 Mean = 180.7
Replicate 2 Mean = 235.2
Frequency (x10-3)
Read Pair Fragment Size (Sox9 CUT&RUNs)
c
pearson's r = 0.80
Adjusted R2 = 0.65
Sox9 CUT&RUN
Consensus Peakset
log10(Sox9 Replicate 2 CPM + 1)
log10(Sox9 Replicate 1 CPM + 1)
d
Sox9 Enrichment Over IgG
Sox9
IgG
-1.0 kb
Center
1.0
e
pearson's r = 0.94
Adjusted R2 = 0.89
ATAC-seq
5kb Genomic Bins
log10(Control MO Replicate 2 Read Count + 1)
log10(Control MO Replicate 1 Read Count + 1)
f
Replicate 1 Mean = 170.4
Replicate 2 Mean = 169.4
Frequency (x10-3)
Read Pair Fragment Size (Control MO ATAC-seq)
g
pearson's r = 0.95
Adjusted R2 = 0.91
ATAC-seq
Consensus Peakset
log10(Control MO Replicate 2 CPM + 1)
log10(Control MO Replicate 1 CPM + 1)
h
pearson's r = 0.93
Adjusted R2 = 0.86
ATAC-seq
5kb Genomic Bins
log10(Sox9 MO Replicate 2 Read Count + 1)
log10(Sox9 MO Replicate 1 Read Count + 1)
i
Replicate 1 Mean = 181.8
Replicate 2 Mean = 170.2
Frequency (x10-3)
Read Pair Fragment Size (Sox9 MO ATAC-seq)
j
pearson's r = 0.97
Adjusted R2 = 0.95
ATAC-seq
Consensus Peakset
log10(Sox9 MO Replicate 2 CPM)
log10(Sox9 MO Replicate 1 CPM)
k
Control MO
Sox9 MO
Principal Component #2 (25%)
Principal Component #1 (62%)
l
TSS Enrichment in Control MO Replicates
Replicate 1
Replicate 2
Library size normalized average ATAC-seq signal
-5.0
TSS
5.0kb
m
TSS Enrichment in Sox9 MO Replicates
Replicate 1
Replicate 2
Library size normalized average ATAC-seq signal
-5.0
TSS
5.0kb

Supplementary Figure 8 – Quality control metrics for SOX9 CUT&RUN and SOX9/Control MO ATAC-seq samples.

(**A**) Scatterplot of read count from each SOX9 replicate CUT&RUN alignment file at regions obtained after binning the genome into 5kb fragments (213311 bins). Magenta line is the line of best fit from regression analysis. (**B**) Overlayed histograms of read pair fragment size distribution for both SOX9 CUT&RUN replicates. (**C**) Scatter plot of sequencing depth normalized SOX9 signal from each replicate at consensus SOX9 peakset (9699 peaks). (**D**) Profile plot showing cumulative SOX9 and IgG signal at SOX9 consensus peakset. SOX9 signal displayed as average between replicates. (**E**) Scatterplot of read count from each Control MO ATAC-seq replicate alignment file at regions obtained after binning genome into 5 kb fragments (213367 bins). Magenta line is the line of best fit from regression analysis. (**F**) Overlayed histograms of read pair fragment size distribution for both Control MO ATAC-seq replicates. (**G**) Scatter plot of sequencing depth normalized ATAC-seq signal from each replicate at consensus Control MO peakset (73479 peaks). (**H**) Scatterplot of read count from each SOX9 MO ATAC-seq replicate alignment file at regions obtained after binning genome into 5 kb fragments (213367 bins). Magenta line is the line of best fit from regression analysis. (**I**) Overlayed histograms of read pair fragment size distribution for both SOX9 MO ATAC-seq replicates. (**J**) Scatter plot of sequencing depth normalized ATAC-seq signal from each replicate at consensus SOX9 MO peakset (84529 peaks). (**K**) Principal Component Analysis (PCA) plot showing replicates of each sample (Control and SOX9 MO). Principal component #1, accounting for most of the variance, separates the samples by treatment (dashed gray lines). (**L – M**) Profile plots showing enrichment of ATAC-seq signal at TSS for both replicates of each sample.

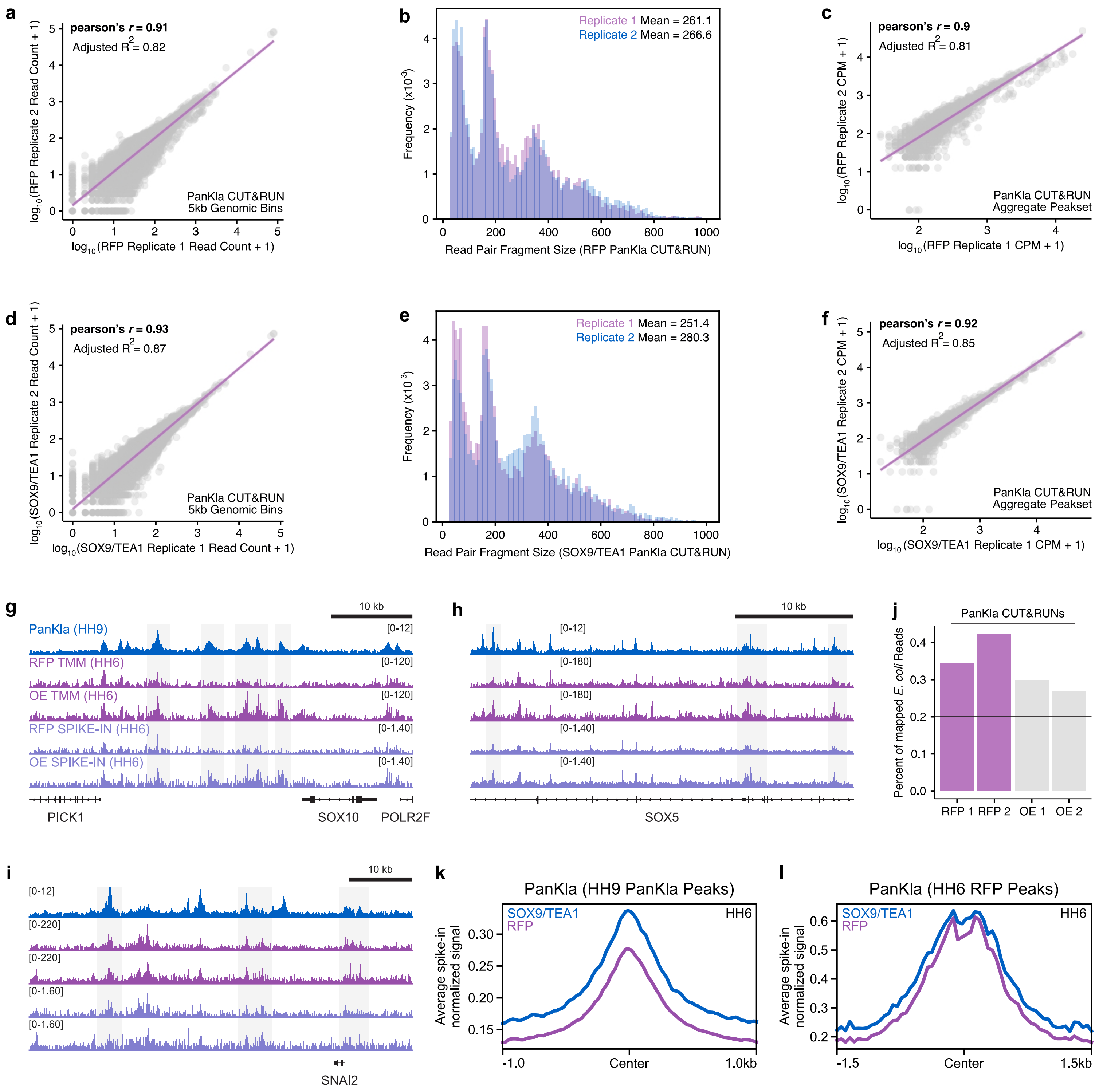
a
pearson's r = 0.91
Adjusted R2 = 0.82
PanKla CUT&RUN
5kb Genomic Bins
log10(RFP Replicate 2 Read Count + 1)
log10(RFP Replicate 1 Read Count + 1)
b
Replicate 1 Mean = 261.1
Replicate 2 Mean = 266.6
Frequency (x10-3)
Read Pair Fragment Size (RFP PanKla CUT&RUN)
c
pearson's r = 0.9
Adjusted R2 = 0.81
PanKla CUT&RUN
Aggregate Peakset
log10(RFP Replicate 2 CPM + 1)
log10(RFP Replicate 1 CPM + 1)
d
pearson's r = 0.93
Adjusted R2 = 0.87
PanKla CUT&RUN
5kb Genomic Bins
log10(SOX9/TEA1 Replicate 2 Read Count + 1)
log10(SOX9/TEA1 Replicate 1 Read Count + 1)
e
Replicate 1 Mean = 251.4
Replicate 2 Mean = 280.3
Frequency (x10-3)
Read Pair Fragment Size (SOX9/TEA1 PanKla CUT&RUN)
f
pearson's r = 0.92
Adjusted R2 = 0.85
PanKla CUT&RUN
Aggregate Peakset
log10(SOX9/TEA1 Replicate 2 CPM + 1)
log10(SOX9/TEA1 Replicate 1 CPM + 1)
g
10 kb
PanKla (HH9) [0-12]
RFP TMM (HH6) [0-120]
OE TMM (HH6) [0-120]
RFP SPIKE-IN (HH6) [0-1.40]
OE SPIKE-IN (HH6) [0-1.40]
PICK1
SOX10
POLR2F
h
10 kb
[0-12]
[0-180]
[0-180]
[0-1.40]
[0-1.40]
SOX5
j
PanKla CUT&RUNs
Percent of mapped E. coli Reads
RFP 1
RFP 2
OE 1
OE 2
i
10 kb
[0-12]
[0-220]
[0-220]
[0-1.60]
[0-1.60]
SNAI2
k
PanKla (HH9 PanKla Peaks)
SOX9/TEA1
RFP
HH6
Average spike-in normalized signal
-1.0
Center
1.0kb
l
PanKla (HH6 RFP Peaks)
SOX9/TEA1
RFP
HH6
Average spike-in normalized signal
-1.5
Center
1.5kb

Supplementary Figure 9 – Quality control metrics for SOX9/TEA1-VPR over-expression CUT&RUN experiment.

(**A**) Scatterplot of read count from each PanKla CUT&RUN performed on pCI-H2B-RFP (Control) replicate samples (transfected HH6 embryos). Read count computed for each alignment file at regions obtained after binning the genome into 5kb fragments (213313 bins). Magenta line is the line of best fit from regression analysis. (**B**) Overlayed histograms of read pair fragment size distribution for PanKla CUT&RUNs performed on pCI-H2B-RFP replicate samples. (**C**) Scatter plot of sequencing depth normalized PanKla signal from each pCI-H2B-RFP replicate at aggregate PanKla peakset from control (RFP) and SOX9/TEA1-VPR over-expression (OE) peaksets (2188 peaks). (**D**) Scatterplot of read count from each PanKla CUT&RUN performed on OE replicate samples (transfected HH6 embryos). Read count computed for each alignment file at regions obtained after binning the genome into 5kb fragments (213313 bins). Magenta line is the line of best fit from regression analysis. (**E**) Overlayed histograms of read pair fragment size distribution for PanKla CUT&RUNs performed on OE replicate samples. (**F**) Scatter plot of sequencing depth normalized PanKla signal from each OE replicate at aggregate PanKla peakset from RFP OE peaksets (2188 peaks). (**G - I**) Genome browser tracks showing the average (between replicates) normalized (spike-in and TMM) signal for RFP and OE samples at the SOX10, SOX5, and SNAI2 loci. Tracks are arranged in the same order as panel G. (**J**) Barplot of the percent of reads from the fastq files associated with the experimental samples that mapped to the *E. coli* genome. These values were used to calculate spike-in normalization of experimental samples as described by Skene et al., 2018 [4] (see Methods section). The fraction of *E. coli* reads in these samples is consistent with previous reports using CUT&RUN assays [4]. (**K**) Profile plot showing the average cumulative spike-in normalized HH6 PanKla CUT&RUN signal of RFP (magenta) and SOX9+TEA1-VPR (purple) samples at HH9 PanKla CUT&RUN consensus peakset. (**L**) Profile plot showing the average cumulative spike-in normalized HH6 PanKla CUT&RUN signal of RFP (magenta) and SOX9+TEA1-VPR (purple) samples at HH6 PanKla CUT&RUN RFP aggregate peakset (2188 peaks). RefSeq gene annotation tracks are used to visualize genes in genome browser panels. Non-curated non-coding RNA annotations are not displayed.
