# Supplementary material for: Histone lactylation couples cellular metabolism with developmental gene regulatory networks
Source: Nat Commun. 2024 Jan 2;15:90. doi: 10.1038/s41467-023-44121-1 (PMC10762033; doi:10.1038/s41467-023-44121-1)
Supplement: Supplementary file 3 — Description of Additional Supplementary Files [file 41467_2023_44121_MOESM3_ESM.pdf]

Description of Additional Supplementary Files

File Name: Supplementary Data 1
Description: Table of flow cytometry data containing PanKla and TFAP2B levels for cells from HH9 embryonic heads.

File Name: Supplementary Data 2
Description: Table of flow cytometry data containing PanKla levels for TFAP2B-positive cells from HH9 and HH12+ embryonic heads as well as for Pax7-positive cells from HH6 whole embryos.

File Name: Supplementary Data 3
Description: Table of consensus PanKla CUT&RUN peakset from HH9 NCCs with closest gene assignment information.

File Name: Supplementary Data 4
Description: Table of GO terms enriched among genes associated with the most highly lactylated peaks from the PanKla CUT&RUN dataset.

File Name: Supplementary Data 5
Description: Table listing the NCC enhancer elements compiled from the literature.

File Name: Supplementary Data 6
Description: Table of consensus H3K18La CUT&RUN peakset from HH9 NCCs with closest gene assignment information.

File Name: Supplementary Data 7
Description: Ranked list of motifs enriched the neural crest scATAC-seq peakset.

File Name: Supplementary Data 8
Description: Table of differential accessibility results for DMSO vs (R)-GNE-140 ATAC-seq samples.

File Name: Supplementary Data 9
Description: Table of differential analysis results for NCC vs PSM PanKla CUT&RUN samples.

File Name: Supplementary Data 10
Description: Table of GO terms enriched among genes associated with NCC- or PSM-enriched PanKla peaks.

File Name: Supplementary Data 11
Description: Table of results obtained from running chromVAR on the NCC vs PSM PanKla differential analysis dataset.

File Name: Supplementary Data 12
Description: Table of results obtained from Nanostring experiment on LDHA/B MO and GTBlue-Control MO transfected neural folds from HH9 embryos.

File Name: Supplementary Data 13
Description: Table of results obtained from running HOMER on the consensus PanKla CUT&RUN peakset.

File Name: Supplementary Data 14
Description: Table of differential accessibility results for GTBlue-Control vs SOX9 MO ATAC-seq samples.
