# Supplementary material for: Histone lactylation couples cellular metabolism with developmental gene regulatory networks
Source: Nat Commun. 2024 Jan 2;15:90. doi: 10.1038/s41467-023-44121-1 (PMC10762033; doi:10.1038/s41467-023-44121-1)
Supplement: Supplementary file 18 — Reporting Summary [file 41467_2023_44121_MOESM18_ESM.pdf]

nature portfolio

Corresponding author(s): Marcos Simoes-Costa

Last updated by author(s): 19 November 2023

Reporting Summary

Nature Portfolio wishes to improve the reproducibility of the work that we publish. This form provides structure for consistency and transparency in reporting. For further information on Nature Portfolio policies, see our Editorial Policies and the Editorial Policy Checklist.

Please do not complete any field with "not applicable" or n/a. Refer to the help text for what text to use if an item is not relevant to your study.
For final submission: please carefully check your responses for accuracy; you will not be able to make changes later.

Statistics

For all statistical analyses, confirm that the following items are present in the figure legend, table legend, main text, or Methods section.

n/a	Confirmed	
☐	☑	The exact sample size (*n*) for each experimental group/condition, given as a discrete number and unit of measurement
☐	☑	A statement on whether measurements were taken from distinct samples or whether the same sample was measured repeatedly
☐	☑	The statistical test(s) used AND whether they are one- or two-sided<br>*Only common tests should be described solely by name; describe more complex techniques in the Methods section.*
☑	☐	A description of all covariates tested
☐	☑	A description of any assumptions or corrections, such as tests of normality and adjustment for multiple comparisons
☐	☑	A full description of the statistical parameters including central tendency (e.g. means) or other basic estimates (e.g. regression coefficient) AND variation (e.g. standard deviation) or associated estimates of uncertainty (e.g. confidence intervals)
☐	☑	For null hypothesis testing, the test statistic (e.g. *F*, *t*, *r*) with confidence intervals, effect sizes, degrees of freedom and *P* value noted<br>*Give P values as exact values whenever suitable.*
☑	☐	For Bayesian analysis, information on the choice of priors and Markov chain Monte Carlo settings
☑	☐	For hierarchical and complex designs, identification of the appropriate level for tests and full reporting of outcomes
☑	☐	Estimates of effect sizes (e.g. Cohen's *d*, Pearson's *r*), indicating how they were calculated

Our web collection on statistics for biologists contains articles on many of the points above.

Software and code

Policy information about availability of computer code

Data collection	BD FACSCelesta Software for flow cytometric analysis, Illumina FASTQ Toolkit for sequencing
Data analysis	fastqc (v0.11.7), cutadapt (v2.10), bowtie2 (v2.42), samtools (v1.7), bedtools (v2.29.2), macs2 (v2.2.7.1), Picard (v2.18.16),wiggletools, Deeptools (v3.5.0), featureCounts (v1.6.2), IGV (v2.13), edgeR (v3.36.0), DiffBind (v2.14.0), CHIPseeker (v1.5.1), biomaRt (v2.50.3), chromVAR (v1.16.0), JASPAR (2020), motifmatchr (v1.16.0), BSgenome.Ggallus.UCSC.galGal6, TFBSTools (v1.32.0), org.Gg.eg.db, clusterProfiler (v4.2.2), GenomicRanges (v1.46.1), FCS Express 7, FIJI (v2.3.0)

For manuscripts utilizing custom algorithms or software that are central to the research but not yet described in published literature, software must be made available to editors and reviewers. We strongly encourage code deposition in a community repository (e.g. GitHub). See the Nature Portfolio guidelines for submitting code & software for further information.

Data

Policy information about availability of data

All manuscripts must include a data availability statement. This statement should provide the following information, where applicable:

- Accession codes, unique identifiers, or web links for publicly available datasets
- A description of any restrictions on data availability
- For clinical datasets or third party data, please ensure that the statement adheres to our policy

All data generated during the current study is available in the GEO repository, GSE228343. This is includes raw data as well as processed/normalized files. Furthermore, wherever applicable, tables with results for genomics, flow cytometry, and gene expression quantification experiments are provided as supplmentary tables.

Research involving human participants, their data, or biological material

Policy information about studies with human participants or human data. See also policy information about sex, gender (identity/presentation), and sexual orientation and race, ethnicity and racism.

Reporting on sex and gender	NA
Reporting on race, ethnicity, or other socially relevant groupings	NA
Population characteristics	NA
Recruitment	NA
Ethics oversight	NA

Note that full information on the approval of the study protocol must also be provided in the manuscript.

Field-specific reporting

Please select the one below that is the best fit for your research. If you are not sure, read the appropriate sections before making your selection.

☑ Life sciences ☐ Behavioural & social sciences ☐ Ecological, evolutionary & environmental sciences

For a reference copy of the document with all sections, see nature.com/documents/nr-reporting-summary-flat.pdf

Life sciences study design

All studies must disclose on these points even when the disclosure is negative.

Sample size	Sample sizes were not pre-determined and are similar to those reported by other studies using similar approaches (Rothstein et al., Genome Research 2019; Azambuja and Simoes-Costa, Dev Cell 2020).
Data exclusions	For the multiple regression analysis with SOX9, YAP1, and PanKla, only PanKla peaks with CPM>0 in each sample/replicate (for all factors) were chosen for analysis. Therefore, 10,448 (out of 10,912) peaks were considered. Rationale for this is provided in the methods section. For the flow cytomtery analysis in Figure 1F, 9 cells (with negative values) were excluded from the HH9 dataset on the basis of being outliers and the square root transformation was performed. Excluded cells made up 0.58% of total cells.
Replication	All genomics experiments were replicated at least twice (two biological replicates). Replication information for other experimetns can be found in the methods section pertaining to a specific experiment. Due to the design of our study the replicates for all experiments are bioligical relicates.
Randomization	In experiments that did not involve a paired design such as SOX9 loss-of-function (Figure 3D) and SOX9/TEA1-VPR over-expression (Figure 3I), healthy wild-type embryos were randomly assigned to control or treatment groups.
Blinding	Blinding was not possible or relevant in our study. GT-Blue Control and FITC-LDHA/B morpholino transfected embryos were subjected to screening for transfection efficiency, thereby reavealing control and treated sides. For flow cytometric measurement of lactylation levels, FITC control and FITC-LDHA/B MO had to be injected on the same respective side in all embryos in order to ensure proper collection of control or treated half embryonic heads for subsequent analysis. Lastly, lactate treated explants were phenotypic upon collection.

Behavioural & social sciences study design

All studies must disclose on these points even when the disclosure is negative.

Study description	
Research sample	
Sampling strategy	
Data collection	
Timing	
Data exclusions	
Non-participation	
Randomization	

Ecological, evolutionary & environmental sciences study design

All studies must disclose on these points even when the disclosure is negative.

Study description	
Research sample	
Sampling strategy	
Data collection	
Timing and spatial scale	
Data exclusions	
Reproducibility	
Randomization	
Blinding	

Did the study involve field work? ☐ Yes ☐ No

Field work, collection and transport

Field conditions	
Location	
Access & import/export	
Disturbance	

Reporting for specific materials, systems and methods

We require information from authors about some types of materials, experimental systems and methods used in many studies. Here, indicate whether each material, system or method listed is relevant to your study. If you are not sure if a list item applies to your research, read the appropriate section before selecting a response.

Materials & experimental systems

n/a	Involved in the study	
☐	☑	Antibodies
☑	☐	Eukaryotic cell lines
☑	☐	Palaeontology and archaeology
☐	☑	Animals and other organisms
☑	☐	Clinical data
☑	☐	Dual use research of concern
☑	☐	Plants

Methods

n/a	Involved in the study	
☑	☐	ChIP-seq
☐	☑	Flow cytometry
☑	☐	MRI-based neuroimaging

Antibodies

Antibodies used

rabbit polyclonal anti-L-Lactyl-lysine (PTM Biolabs, PTM-1401 polyclonal)
rabbit polyclonal anti-H3K18La (PTM Biolabs, PTM-1406 polyclonal)
rabbit monoclonal anti-Sox9 (Millipore, AB5535)
rabbit monoclonal anti-Caspase3 (R&D Systems, AF835)
rabbit monoclonal anti-pH3 (Cell Signaling, 9701S)
mouse monoclonal anti-AP2 beta (Santa Cruz Biotechnology, sc-390119)

Validation

Lactylation antiobodies have been validated by the manufacturer (PTM Biolabs). Furthermore, we have performed pan lactylation levels using flow cytometry upon LDHA/B knock-down and observed a decrease in the population of cells that have high lactylation levels. We have also observed dynamic regulation of lactylation levels at differetn stages of NCC development that are consistent with the metabolic transitions udergone by these cells (see Figure 1F and Supplemental Figure 1). AP2B antibody was validated by Rothstein and Simoes-Costa (Genome Research, 2019). Caspase3, pH3, and SOX9 antibodies exhibit expected staining patterns.

Eukaryotic cell lines

Policy information about cell lines and Sex and Gender in Research

Cell line source(s)	
Authentication	
Mycoplasma contamination	
Commonly misidentified lines (See ICLAC register)	

Palaeontology and Archaeology

Specimen provenance	
Specimen deposition	
Dating methods	

☐ Tick this box to confirm that the raw and calibrated dates are available in the paper or in Supplementary Information.

Ethics oversight	

Note that full information on the approval of the study protocol must also be provided in the manuscript.

Animals and other research organisms

Policy information about studies involving animals; ARRIVE guidelines recommended for reporting animal research, and Sex and Gender in Research

Laboratory animals	This study did not use laboraty animals.
Wild animals	This study did not use wild animals.
Reporting on sex	This was not relevant in our study
Field-collected samples	This study did not use any field-collected samples.
Ethics oversight	According to the Office for Protection from Research Risks (OPRR) the term "live vertebrate animal" applies to avians (such as chicken embryos) after hatching. All embryos used in this study were not incubated longer than 5 days.

Note that full information on the approval of the study protocol must also be provided in the manuscript.

Clinical data

Policy information about clinical studies

All manuscripts should comply with the ICMJE guidelines for publication of clinical research and a completed CONSORT checklist must be included with all submissions.

Clinical trial registration	
Study protocol	
Data collection	
Outcomes	

Dual use research of concern

Policy information about dual use research of concern

Hazards

Could the accidental, deliberate or reckless misuse of agents or technologies generated in the work, or the application of information presented in the manuscript, pose a threat to:

No | Yes

- [ ] [ ] Public health
- [ ] [ ] National security
- [ ] [ ] Crops and/or livestock
- [ ] [ ] Ecosystems
- [ ] [ ] Any other significant area

Experiments of concern

Does the work involve any of these experiments of concern:

No | Yes

- [ ] [ ] Demonstrate how to render a vaccine ineffective
- [ ] [ ] Confer resistance to therapeutically useful antibiotics or antiviral agents
- [ ] [ ] Enhance the virulence of a pathogen or render a nonpathogen virulent
- [ ] [ ] Increase transmissibility of a pathogen
- [ ] [ ] Alter the host range of a pathogen
- [ ] [ ] Enable evasion of diagnostic/detection modalities
- [ ] [ ] Enable the weaponization of a biological agent or toxin
- [ ] [ ] Any other potentially harmful combination of experiments and agents

Plants

Seed stocks

Novel plant genotypes

Authentication

ChIP-seq

Data deposition

- [ ] Confirm that both raw and final processed data have been deposited in a public database such as GEO.
- [ ] Confirm that you have deposited or provided access to graph files (e.g. BED files) for the called peaks.

Data access links
May remain private before publication.

Files in database submission

Genome browser session
(e.g. UCSC)

Methodology

Replicates

Sequencing depth

Antibodies

Peak calling parameters

Data quality

Software

Flow Cytometry

Plots

Confirm that:

- [x] The axis labels state the marker and fluorochrome used (e.g. CD4-FITC).
- [x] The axis scales are clearly visible. Include numbers along axes only for bottom left plot of group (a 'group' is an analysis of identical markers).
- [x] All plots are contour plots with outliers or pseudocolor plots.
- [x] A numerical value for number of cells or percentage (with statistics) is provided.

Methodology

Sample preparation: See Methods section "Cell Suspension Staining of Embryonic Tissue for Flow Cytometry"

Instrument: BD FACSCelesta

Software: FCS Express 7

Cell population abundance:

Gating strategy:

For timecourse flow experiment -
HH6 replicates - at least 6000 single cells (FCS-H vs FSC-A) and at least 2000 PAX7+ cells were analyzed
HH9 replicates - at least 17,000 single cells and at least 1,500 AP2B+ cells were analyzed
HH12 replicates - at least 7,000 singel cells and at least 2,000 AP2B+ cells were analyzed
Gates are the same and set based on the secondary antibody control for all samples. Secondary antibodies for PAX7 and AP2B antuobodies are the same.
For PanKla measurement upon LDHA/B knockdown -
FITC Control MO - 3,305 single cells and 671 FITC+ cells were analyzed
FITC LDHA/B MO - 3,168 single cells and 477 FITC+ clls were analyzed

- [x] Tick this box to confirm that a figure exemplifying the gating strategy is provided in the Supplementary Information.

Gating Strategy - SSC-A vs FSC-A >> FSC-H vs FSC-A >> gating of fluorescent channels based on unstained and/or secondary antibody only controls

Magnetic resonance imaging

Experimental design

Design type:

Design specifications:

Behavioral performance measures:

Imaging type(s):

Field strength:

Sequence & imaging parameters:

Area of acquisition:

Diffusion MRI: ☐ Used ☐ Not used

Preprocessing

Preprocessing software:

Normalization:

Normalization template:

Noise and artifact removal:

Volume censoring:

Statistical modeling & inference

Model type and settings:

Effect(s) tested:

Specify type of analysis: ☐ Whole brain ☐ ROI-based ☐ Both

Statistic type for inference

(See Eklund et al. 2016)

Correction

Models & analysis

n/a	Involved in the study
☐	☐ Functional and/or effective connectivity
☐	☐ Graph analysis
☐	☐ Multivariate modeling or predictive analysis

Functional and/or effective connectivity

Graph analysis

Multivariate modeling and predictive analysis

This checklist template is licensed under a Creative Commons Attribution 4.0 International License, which permits use, sharing, adaptation, distribution and reproduction in any medium or format, as long as you give appropriate credit to the original author(s) and the source, provide a link to the Creative Commons license, and indicate if changes were made. The images or other third party material in this article are included in the article's Creative Commons license, unless indicated otherwise in a credit line to the material. If material is not included in the article's Creative Commons license and your intended use is not permitted by statutory regulation or exceeds the permitted use, you will need to obtain permission directly from the copyright holder. To view a copy of this license, visit http://creativecommons.org/licenses/by/4.0/
